# How to teach the anatomy of the inguinal canal? A multimodal approach

**DOI:** 10.1016/j.heliyon.2025.e42434

**Published:** 2025-02-04

**Authors:** Shina Yeo Qing Chun, Nathaniel Dexter Salim, Lai YuFu Jarrell, Joshua Koh Zhi Yuan, Eng-Tat Ang, Davide Lomanto

**Affiliations:** a Dept. of Anatomy, Yong Loo Lin School of Medicine, NUS, National University of Singapore, Singapore; b Dept. of Surgery, Yong Loo Lin School of Medicine, NUS, National University of Singapore, Singapore; c Duke NUS medical School, National University of Singapore, Singapore

**Keywords:** Inguinal canal, Anatomy teaching, Model creation, Didactic lectures, Group learning, Individual learning, Collaborative learning

## Abstract

**Introduction:**

Learning the anatomy of the inguinal canal is challenging for medical students and residents. Current teaching via didactic lectures may not suffice for optimal learning, and visualization of the complicated 3D-structure of the inguinal canal. Hence, we investigated the efficacy of a low-fidelity model creation to improve the teaching.

**Materials and methods:**

30 pre-clinical students were split into three groups and assigned different teaching interventions: 1) Lecture (Group L), 2) Lecture with individual model creation (Group M1), and 3) Lecture with group model creation (Group M2). Gain in knowledge were assessed through a pre- and post-intervention multiple-choice quizzes (MCQs), while perceptions of confidence/engagement/model efficacy were measured using post-intervention questionnaires.

**Results:**

For knowledge acquisition, average MCQ scores improved most for Group L (23 ± 1%), followed by M1 (19 ± 1%) and M2 (13 ± 1%). In learning the inguinal canal, perceived confidence was enhanced by modelling compared to lecture only (p < 0.05). There was no difference between group M1 and M2 respectively. Perceived engagement had also been enhanced by modelling compared to lecture only (p < 0.05). Interestingly, there was statistical difference between M1 and M2 (p < 0.05). However, perceived usefulness of the model compared to lecture only was insignificant (p > 0.05). There was also no difference between M1 and M2 (p > 0.05).

**Conclusion:**

As a supplement to didactic teaching of the inguinal canal, model creation was well-received, and provided opportunities for experiential learning. However, the usefulness of modelling in learning the anatomy and pathologies of inguinal canal was insignificant. Our research design was inadequate in showing long term gain. Additionally, results from group model creation could be affected by the dynamics amongst members. Future work should aim to address these issues.

## 1 Introduction

In Singapore, inguinal hernias are one of the most common surgical pathologies. In 2010, Tan Tock Seng Hospital, a tertiary hospital in Singapore, reported a total of 561 repairs being performed [1]. Given the prevalence, medical students in the National University of Singapore, Yong Loo Lin School of Medicine (NUS-YLLSoM) are taught the inguinal canal, and hernia pathologies in the first-year curriculum. However, these students including surgical trainees frequently experienced difficulties in understanding the inguinal canal anatomy, possibly due to the region's inherent complexity [2].

Such challenges might also be compounded by the existing non-ideal teaching modalities. In NUS-YLLSoM, content relevant to inguinal canal anatomy and pathology were taught mostly through didactic lectures to a large audience of 280 students. These activities are usually accompanied by learning materials (*e.g.*, slide, textbooks). Often, students would annotate the provided learning materials during the presentation, underscoring the lack of interaction, and over reliance on passive learning (observation, listening, reading). This resulted in low engagement and poor memory of teaching material [3]. As remediation, the collaborative learning cases (CLC) teaching pedagogy was initiated at the NUS-YLLSoM, resembling problem-based learning (PBL) utilised in medical schools worldwide [4]. Teachings occurred in groups of six, with a maximum of 30 students per session. Before the class, students were required to do some pre-reading and a pre-quiz to prepare for the group discussions. These students would solve complex, open-ended problems detailing various clinical cases. After the class, students and the CLC facilitators would then engage in a Q&A session to clarify doubts and consolidate the knowledge. Current literature seems to indicate that small-group learning provides a social dimension which improves support, and sense of responsibility amongst students [4,5].

To optimised learning of anatomy, “Silent mentors” (i.e. respectful term for cadavers) and virtual reality were utilised to enhance learning experiences for medical students [[6], [7], [8]]. These have resulted in improvement to students’ understanding, technical mastery, and satisfaction with learning [9].

The use of low-fidelity inguinal canal models, constructed from basic materials have also shown multiple benefits. Such include reducing the cognitive load associated with learning novel topics and increasing eagerness towards learning [10,11]**.** Additionally**,** there is improved spatial awareness of the inguinal canal, and longer-term retention of anatomical knowledge. These models could also incorporate a demonstration of repair through the application of mesh, seen in the transabdominal preperitoneal (TAPP) and totally extraperitoneal (TEP) procedures of inguinal hernia repair [[12], [13], [14]]**.** We believe that our current research would contribute more to the discussion. No similar study has been done in the context of an Asian population. We also believe our detailed model allows for greater understanding of the inguinal anatomy, pathology, and repair of hernias. Both translucent tracing and coloured paper were utilised to demonstrate the order, and relationships of the various layers in the abdominal wall. Furthermore, it includes surgical landmarks and that allows for differentiation of posterior versus anterior approaches to hernia repair. In essence, our model affords surgical training which bridges pre-clinical and clinical knowledge. Importantly, hernia specialists were consulted during the planning to ensure accuracy in terms of anatomy, and surgical techniques in laparoscopic repair. Finally, our research also examines how group dynamics may interfere with the learning process.

In summary, given the current state of curriculum here in Singapore, we hypothesized that the combined use of didactic teaching and a low-fidelity model would encourage more experiential learning. We further hypothesized that individual modelling endeavour would be more productive than group work.

Therefore, this study aims to.

1. Evaluate the pre-clinical students' level of understanding of the anatomy and pathology of the inguinal canal.
2. Compare the effectiveness and satisfaction of using a low-fidelity model with didactic lectures against didactic lectures alone in inguinal canal teaching.
3. Evaluate the effectiveness and satisfaction of the low-fidelity model when taught in group versus individual setting.

## 2 Materials and methods

**Fig. 1:**
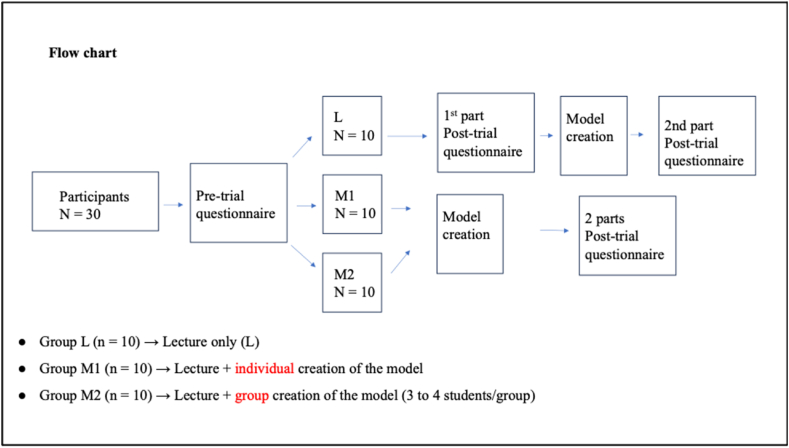
Flowchart of learning interventions.

Pre-clinical medical students (n = 30) from NUS-YLLSoM were recruited to participate in the trial. To qualify for the study, the selected students must have had prior exposure to the basic inguinal anatomy and hernia pathology curriculum, but no prior exposure to the clinical learning of inguinal hernia management in the surgical theatre. The students were randomly assigned into 3 groups (n = 10). The groups had to first complete the same pre-trial questionnaire, and didactic lecture. They were then assigned to different learning interventions (See Fig. 1). This project has been evaluated and approved by the NUS IRB (Reference code: NUS-IRB-2022-589).

### 2.1 Pre-trial questionnaire

All participants were required to complete a pre-trial questionnaire consisting of two sections (See Appendix A, Appendix A). The first section specifically detailed 6 questions regarding participants’ opinions on the inguinal anatomy education provided by the school (10-point Likert scale). The second section detailed 10 multiple-choice questions (MCQs) evaluating their pre-trial knowledge of the inguinal canal anatomy and hernias. The mean score for each group was then calculated for pre-post comparison.

The 10 questions were.

1) What forms most of the anterior wall of the inguinal canal?
2) Where is the deep inguinal ring located?
3) Which direction does the inguinal canal travel?
4) Which artery DOES NOT run in the inguinal canal?
5) What DOES NOT run through the inguinal canal in females?
6) Which is the MOST common hernia in females?
7) Fill in the blanks: Indirect inguinal hernias pass ____ to the inferior epigastric vessels.
8) Which of the following DO NOT make up the boundaries of the Hesselbach's Triangle?
9) What defines a direct inguinal hernia?
10) What is the current recommended management for most male patients with bilateral inguinal hernias?

### 2.2 Didactic lecture

The 45-min lecture include 1) Boundaries of inguinal canal, 2) Canal contents in males versus females, 3) Surgical landmarks in inguinal canal anatomy, 4) Types of inguinal hernias, and 5) Methods of differentiating direct versus indirect inguinal hernias. Additionally, management of inguinal hernias through laparoscopic (TEP, TAPP) versus open surgery were taught (absent in current curriculum). The lecture was taught by a senior medical student, but the contents covered were vetted and approved by the faculty members specializing in inguinal canal anatomy, and hernia repairs.

### 2.3 Model creation

In this 1-h session, participants were provided with the materials and equipment (See Appendix 3) to create a low-fidelity model of the inguinal canal, accompanied with a step-by-step instructional video (https://www.youtube.com/watch?v=_GKLS46oWDA), and manual (See Appendix 4). An instructor was present during the session to provide technical assistance, and to answer participant's questions (i.e., which areas to cut out, how long should the marker line be drawn), but was specifically instructed not to answer any questions pertaining to the subject matter.

Once all participants in the group had completed their models, they were guided to orientate it anatomically using knowledge covered in the lecture. A completed model was also displayed for referencing purposes.

### 2.4 Post-trial questionnaire

The two-parts post-trial questionnaire assessed the learning experience through didactic lectures versus low-fidelity model creation (individual or group). After the lecture, Group L completed the first part of the post-trial questionnaire, then partook in model creation before completing the second part of the questionnaire. Group M1 and M2 completed both parts in one setting (See Fig. 1).

The first part consisted of three components (See Appendix 2 and 5). In the first component, a repeat of the same 10 MCQs identical to the pre-trial questionnaire was carried out. This is to evaluate any changes in participants' knowledge following their assigned learning intervention. For each question, the proportion answered correctly by each group was recorded to compare pre-intervention and post-intervention results. In the second component, the “*Opinion on Today's Learning Session*” has 7 questions measuring participants' perceived confidence towards the inguinal anatomy knowledge after intervention. In the third component, 4 questions measured participants' perceived engagement during the teaching session. The second part consisted of two further components. The first “*Opinion on Model-Creation Session*” (See Appendix 6) detailed 6 questions which evaluated the perceived usefulness of model creation as a supplement to the lecture. The second “*Open-ended Responses*” asked two further questions to elicit open-ended feedback for improving the lecture and model creation interventions. Specifically, students were asked via the survey monkey platform “*How could our lecture be improved?” and “How could our model creation session be improved?” respectively. These comments were subsequently edited for clarity and recorded by a member of the team into the Excel spreadsheet for publication purposes*.

#### 2.4.1 Statistics

As the sample sizes were small, the nonparametric tests were used throughout the study. For all the questions, the median and standard deviations were calculated for each intervention group. Each set of questions measured a different variable (perceived confidence, perceived engagement, model effectiveness). For the Likert score data, the Kruskai-Wallis test was performed to compare the three intervention groups (L vs M1, L vs M2, M1 vs M2). The level of significance was set to be 0.05. Additionally, the Wilcoxon test was also performed between the pre- and post-results. The raw data that supported the findings of this study were openly available at http://doi.org/10.6084/m9.figshare.24649260.

## 3 Results

### 3.1 Opinions on inguinal hernia education

**Fig. 2:**
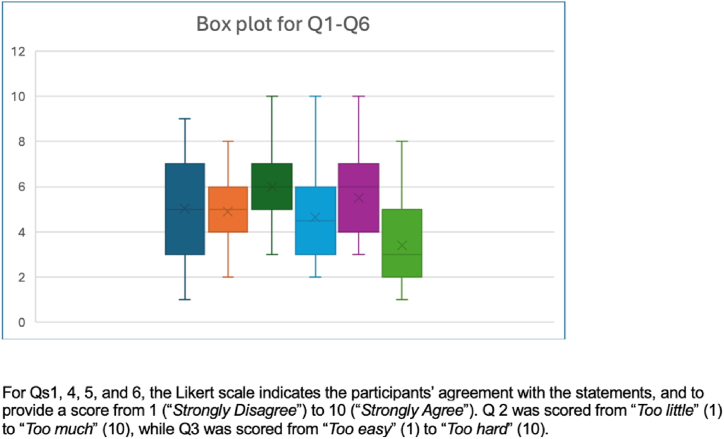
Box plot on the “Opinion of inguinal hernia education”.

For each question, the mean score, median score, and standard deviation were obtained for the entire study (n = 30) (Table 1 and Fig. 2). It was apparent from question 6 that students did not believe that given their current knowledge of the inguinal canal, they would be able to understand the different surgical procedures such as TEP and TAPP.

**Table 1:** The 6 questions regarding participants’ opinions on the inguinal anatomy education provided by the school.

Question	1) *I enjoyed learning about the inguinal anatomy.*	2) *How would you rate the workload designated to inguinal anatomy?*	3) How would you rate the difficulty of the content for inguinal anatomy?	4) *I can still remember the content taught for inguinal anatomy.*	5) *I can identify important landmarks of the inguinal region.*	6) *I believe with my pre-clinical knowledge of the inguinal anatomy; I can understand surgical procedures in repairing hernias.*
**Mean**	5.03	4.90	6.00	4.63	5.50	3.40
**Median**	5	5	6	5	6	3
**S.D.**	2.09	1.40	1.49	1.97	1.81	1.98

### 3.2 Questions on inguinal canal anatomy/hernias

**Fig. 3:**
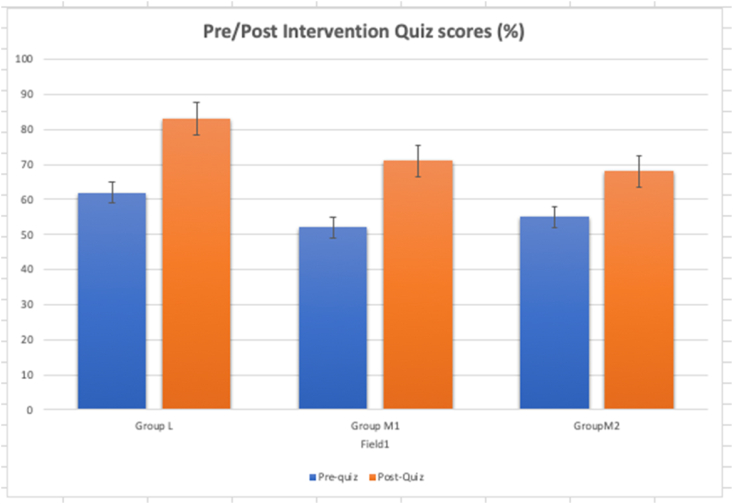
Bar graph of Group L, M1 and M2's pre and post intervention quiz scores.

For each group, the difference in the scores for pre- and post-intervention were calculated (See Fig. 3). It was obvious that the medical students did better in the 10 MCQs post interventions with lectures and modelling (p < 0.05).

### 3.3 Opinion on current learning session and model-creation session

**Fig. 4:**
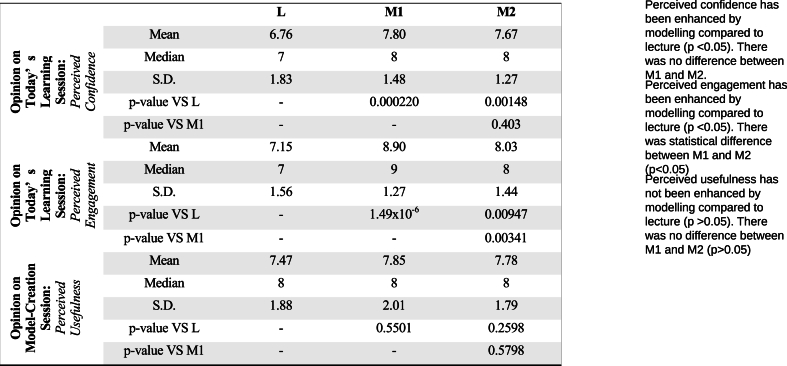
Perceived confidence, perceived engagement and perceived usefulness of teaching modalities.

The mean scores, standard deviations and median scores were presented for each intervention group (See Fig. 4). All values (except median) have been rounded off to three significant figures.

For perceived confidence, Group M1 has the highest overall score followed by M2, then L. The Kruskal-Wallis test showed significant differences (between the median scores of M1 vs L (p = 0.000220) and M2 vs L (p = 0.00148). No significant difference was observed between the median scores of M1 vs M2 (p = 0.403).

For perceived engagement, a similar trend was observed. Group M1 has the highest overall average score followed by M2, then L. The Kruskal-Wallis test showed significant differences between M1 vs L (p = 1.49 x 10^6^), M2 vs L (p = 0.00341), and M1 vs M2 (p = 0.00947).

For perceived usefulness of the model, M1 had the highest overall score for usefulness, followed by M2, then L. The Kruskal-Wallis test showed no significant differences were observed between the overall median scores of all three groups.

As a measure of reliability, Cronbach's Alpha was calculated for each of the three question sets. This yielded alpha coefficients of ɑ = 0.858 (perceived confidence), ɑ = 0.718 (perceived engagement), and ɑ = 0.8859 (model's effectiveness), indicating good internal consistency for all three sets.

### 3.4 Qualitative comments on lecture and model creation session

The following were the most notable ad hoc comments made by the students (76 % of the 30 students responded) after the research. Typically, these comments were quite brief and averaged about 10–12 words (See Table .2).

**Table 2:** Qualitative comments from the participants concerning the lecture and modelling.

**Lecture**
**Comments**
**Summary**
Participants requested more visual analysis of the inguinal canal
Participants expressed desire for more comprehensive content coverage.
Participants also highlighted areas for improvement to keeping them engaged.
“*Images in slides can be enlarged, and more time could've been given to internalize and orientate images, especially those of the laparoscopic view.*”
“*Perhaps you can use 3D imagery (e.g., Complete Anatomy) to help in explaining during the lecture.”*
“*Please explain more about TAPP vs TEP.*”
“*Please cover more terms that were used.*”
“The lecture can be more engaging, like a question-answer kind of thing.”
“Can employ more active recall and engagement techniques.”
“More interactive - easy to zone out.”
“Less wordy.”
**Model Creation**
**Comments**
**Summary**
Participants highlighted that the model creation process was too complex for optimal learning.
Some participants stated that the manual was confusing without the video, while others said vice versa.
“Simpler & fewer steps.”
“Less complicated model.”
“Have a physical final product to refer while creating the model.”
“Make the pieces pre-cut so only assembly is required.”
“Markings on tracing paper could've been done in pencil first so any mistakes can be erased, creating a neater-looking final product.”
“Some instructions could be a bit confusing without the instructional video!
“Please slow down the video as it was sometimes quite hard to follow.”
“Very helpful that the instructions were repeated after the video. Video alone was a bit fast and confusing.”

## 4 Discussion

Our research wanted to assess the effectiveness of using a low-fidelity model to supplement didactic teaching of the anatomy of the inguinal canal, and hernia repair. Interestingly, we also discovered some significant trends in terms of perception, engagement, and confidence in the various teaching interventions. The following paragraphs would discuss our research findings.

### 4.1 Opinions on inguinal hernia education

From the results of the students’ opinions, they had enjoyed learning it with some recollections and identification of the key landmarks in the inguinal region. However, they had low confidence in understanding relevant surgical procedures such as TEP and TAPP. They had rated the subject matter as difficult but thought the designated workload was manageable (See Fig. 2). These results suggested that the inguinal anatomy was a difficult topic for students. The traditional teaching methods did not enable them to have absolute mastery over the content. Hence, reinforcing the need for improved teaching methods.

### 4.2 Lecture vs model learning

From the pre and post quiz, average scores increased most for Group L (23 %), followed by M1 (19 %), and M2 (13 %) (See Fig. 3). These results suggested that lectures were the most effective teaching modality in improving knowledge gain. This undermined the utility of model creation. It did not support our hypothesis that model creation helps to navigate the complexities of the inguinal canal. However, further analysis of the results showed that students in Groups M1 and M2 had significantly higher perceived confidence of their knowledge of the inguinal region compared to L. In addition, Groups M1 and M2 also reported significantly higher perceived engagement than L (See Fig. 4), Collectively, this suggest that there was improved focus, and enjoyment towards model creation. Overall, the perceived usefulness of model creation session was positive with no differences between the groups.

In fact, model creation could be detrimental to the learning process. It could lead to more confusion amongst the learners, without further explanations by the instructors. Importantly, the gap between 2D and 3D must be bridged effectively. Ample time must be provided to examine the model, make the connections, and to clarify deeper concepts. In our research, the difference in perceived confidence/engagement in groups M1 or M2 versus L strongly suggested that model creation was a more impactful and memorable learning experience (see Fig. 4). In terms of engagement, the model creation was an active experiential learning experience. Students found themselves more focused during the model creation compared to passive learning in lectures. Ultimately, we believe that complex anatomy should be taught with higher engagement to facilitate increased retention, motivation, and long-term interest.

### 4.3 Individual vs group model making

Individual model creation (M1) appeared to be more effective than group-based model creation (M2). For the pre-post quiz, group M1 showed better scores for the knowledge gain compared to M2. In addition, the average score for perceived engagement was higher for M1, although this was not statistically significant. It is speculated that the poorer outcomes for M2 could be due to social dynamics. The phenomenon of social loafing could have taken place when disinterested members participated less [15]. The sucker effect, and the feelings of being exploited could have resulted in some students losing interest and therefore contribute less [16]. Some students might find collaboration detrimental towards their learning. A study demonstrated how confident and extroverted students could dominate the proceedings, and overshadow their quieter peers [17]. Similarly, a previous study had suggested that small-group learning might be beneficial in model creation [18]. On the contrary, it was found that team-based learning was more positively received. The study demonstrated that low-performing students assigned to team learning showed greater improvement for a statistics examination compared to those who studied individually [19]. It was proposed that collaborative work allowed more discussion, sharing of knowledge, and reduced the cognitive load associated with learning complex concepts [20,21].

### 4.4 Qualitative comments

Some students suggested a lack of engagement during the lectures and proposed more active learning. Furthermore, some highlighted the importance of using appropriate visual aids such as images/animations which could be helpful in orientating the layers of the inguinal canal. For the modelling work, it was suggested that the number of steps were excessive, and the instructions provided were complicated. This could have affected the utility of having a model.

## 5 Conclusions

Our research showed that didactic teaching increased the students’ knowledge more than modelling, at least in the short-term. Nonetheless, our low-fidelity model was well accepted by the students and was an engaging tool that supplemented didactic teaching. Lastly, individual model-making seemed to be more effective than group model-making, probably because the latter would require more dynamics.

## CRediT authorship contribution statement

**Shina Yeo Qing Chun:** Writing – original draft, Project administration, Data curation, Conceptualization. **Nathaniel Dexter Salim:** Writing – original draft, Methodology. **Lai YuFu Jarrell:** Writing – original draft, Project administration, Data curation. **Joshua Koh Zhi Yuan:** Writing – original draft, Validation, Methodology, Formal analysis, Data curation. **Eng-Tat Ang:** Writing – review & editing, Supervision. **Davide Lomanto:** Supervision.

## 6 Limitations and future work

The small sample size in each group was a major limitation in this research. Efforts should have been made to calculate the optimal sample size required to draw more informative conclusions. In addition, randomization alone might not have addressed balance in academic competencies across groups, and this could be a confounder. It would have been better to have the CGPA data from the onset. The potential impact of the instructor's inexperience could have confounded the results as well. Lastly, demographic variables such as age, gender, etc. should been investigated and compared in the three groups. In terms of the statistics, a pilot study or a separate, representative sample should be used to test Cronbach's alpha to ensure that the reliability estimate was not affected in the study design. These factors should be earnestly considered for all future work and analysis.

## Data availability statement

All raw data are available for review when requested by readers and the journal.

## Declaration of competing interest

The authors declare that they have no known competing financial interests or personal relationships that could have appeared to influence the work reported in this paper.

## Footnotes

Appendix A Supplementary data to this article can be found online at https://doi.org/10.1016/j.heliyon.2025.e42434.
