# Supplementary material for: How to teach the anatomy of the inguinal canal? A multimodal approach
Source: Heliyon. 2025 Feb 4;11(3):e42434. doi: 10.1016/j.heliyon.2025.e42434 (PMC11849626; doi:10.1016/j.heliyon.2025.e42434)
Supplement: Multimedia component 1 [file mmc1.docx]

**Appendices**

**Appendix 1 – Pre-trial questionnaire: Opinions on Inguinal Hernia Education**

1. *I enjoyed learning about the inguinal anatomy.* ***(average: teaching perception)***
2. *How would you rate the workload designated to inguinal anatomy?* ***(average: teaching perception)***
3. ***(average: teaching perception)***
4. *I can still remember the content taught for inguinal anatomy.* ***(average: memory and knowledge)***
5. *I can identify important landmarks of the inguinal region.* ***(average: memory and knowledge)***
6. *I believe with my pre-clinical knowledge of the inguinal anatomy; I can understand surgical procedures in repairing hernias.* ***(low: feelings of ill-preparedness for M3)***

.

**Appendix 2 – Questions on Inguinal Canal Anatomy/Inguinal Hernias**

The 10 questions:

1. *What forms most of the anterior wall of the inguinal canal?*
2. *Where is the deep inguinal ring located?*
3. *Which direction does the inguinal canal travel?*
4. *Which artery DOES NOT run in the inguinal canal?*
5. *What DOES NOT run through the inguinal canal in females?*
6. *Which is the MOST common hernia in females?*
7. *Fill in the blanks: Indirect inguinal hernias pass ____ to the inferior epigastric vessels.*
8. *Which of the following DO NOT make up the boundaries of the Hesselbach's Triangle?*
9. *What defines a direct inguinal hernia?*
10. *What is the current recommended management for most male patients with bilateral inguinal hernias?*

**Appendix 3 – Equipment Used for Low Fidelity Model Making**

**
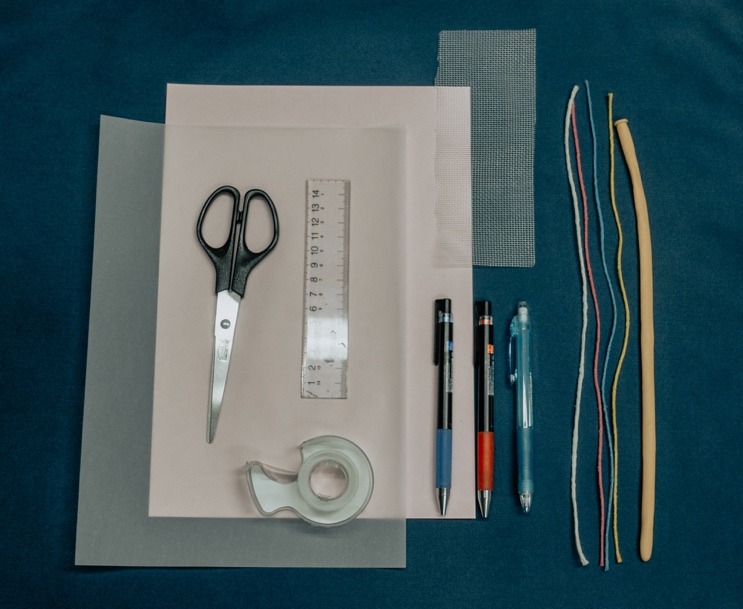
**

List of equipment used: (Left to right) Tracing paper, Coloured paper, Scissors, Ruler, Scotch Tape, Net, Red and Blue pens, Pencil, Various Strings (White, Red, Blue, Yellow), Long rubber balloon.

**Appendix 4 – Manual for Low Fidelity Model Making**

**Instructional Manual**

***Please refer to the instructional video for visual guidance.***

1. Starting from the base, create 4 small markings along the left length of the tracing paper: base to point A (7.5 cm), point A to B (7.5 cm), point B to C (4.5 cm), point C to D (9 cm).
2. Fold the tracing paper along points A, B, C and D. **Refer to the video for the direction of folding**.
3. Make a mark 3.5 cm above point A, label it A1, 5 cm to the right of point A, label it A2, and 2 cm below A2, label it A3. Draw a quadrant joining A1, A2 and A3.
4. Make a mark 2.5 cm above point A, label it as A4, 4 cm to the right of point A, label it as A5, and 2 cm below A5, label it as A6. Draw a quadrant joining A4, A5, and A6, and label the resulting space between the two quadrants as the **pubic arch**.
5. From the outer quadrant and along the fold on point A, draw two parallel lines 0.5 cm between each other, extending to the right length. Label this as the **inguinal ligament**. Where the ligament meets the right length, mark this point as the **Anterior Superior Iliac Spine (ASIS)**.
6. Mark the midpoint of the inguinal ligament (approximately 8 cm from ASIS). 1 cm above this point, draw a small circle (1.5 cm in diameter) and label this as the **deep inguinal ring (DIR**). Using scissors or a pen knife, carefully excise the circle.
7. Mark a point 11.5cm to the right of point B, Label it B1, draw a line joining B1 and A2.
8. Where the diagonal line meets the fold at point B, draw two parallel columns: the left one in blue and the right one in red. The columns should be about 0.3 cm wide, horizontally spaced 0.3 cm from each other, and descend 2 cm below the inguinal ligament. Label these as the **inferior epigastric vessels (IEVs, blue: vein, red: artery)**. Draw over the inguinal ligament.
9. Using the same colour pen, draw two downwards rightwards columns towards the base.  They must be thicker than the IEVs. Label these as the external iliac vessels (EIVs, blue: vein, red: artery)
10. Fill in the IEVs and EIVs with their respective outline colours.
11. Draw a crescent directly left to the EIVs, and label this as the **femoral canal (FC)**. Since it is a pseudo-space, you do not need to excise this region.
12. Cut out a 21 cm by 4.5 cm section of coloured paper, label it as the **arching fibres of the internal oblique and transversus abdominis**. Use tape to stick it underneath the entire region between points B and C.
13. Fold the tracing paper inwards on point B, then on point C. Bring point D to point A to create a triangular cavity. This demonstrates how the inguinal ligament creates the floor of the inguinal canal.
14. Observe where the pubic arch can be seen through the region between points C and D, and trace the quadrant without extending beyond point D. You may gently flatten the sheet for assistance but take care not to create an unwanted fold. Label this as the **pubic arch**.
15. Draw a small circle (1.5 cm in diameter) directly left of the traced pubic arch and label it as the **superficial inguinal ring (SIR)**. Unfold the tracing paper. Using scissors or a pen knife, carefully excise the circle.
16. Cut out a 10 by 9 cm section of coloured paper and label this as the **internal oblique aponeurosis**. Use tape to stick it underneath the right-most region between points C and D. On the region not backed by coloured paper, label it as the **external oblique aponeurosis**.
17. Cut out a 7.5 by 5 cm section of coloured paper and trace the outline of the outer quadrant making up the original pubic arch between points A and B. Cut out the traced area, label the remaining as the **conjoint tendon**, and stick the remaining section underneath the left-most region between points A and B.
18. Label the region between the conjoint tendon and diagonal line drawn step 7 as **Rectus Abdominis muscle**, and label the region enclosed by the rectus, inguinal ligament and IEVs as the **Hesselbach's Triangle**.
19. Thread the red, blue, yellow, and white string into the deep inguinal ring and out of the superficial inguinal ring.
20. Optionally, you may also thread the long balloon through, with the nipple exiting out of the superficial inguinal ring. This represents a section of the small intestine, and shows what happens during an indirect inguinal hernia.
21. Refold the tracing the paper similar to step 15 and tape it down, finalizing the structure.
22. On the plane where the bundle of strings exit the deep inguinal ring, extend the white string over the EIVs and tape it down; label this as the **Vas deferens**.
23. Extend the red and blue string parallel to the EIVs and tape it down ; label this as the **spermatic vessels**.
24. Extend the yellow string towards the lower right corner and tape it down; label this as the **nerve**.
25. Your model is complete!

The region enclosed by the Vas deferens and spermatic vessels is the **triangle of doom**, whereas the region enclosed by the spermatic vessels and inguinal ligament is the **triangle of pain**.

Surgically, the laparoscopic repair of an inguinal hernia involves retracting the hernia back to the abdominal cavity and reinforcing the abdominal wall with a mesh placed on the transversalis fascia.

**Appendix 5 – Opinion on Today’s Learning Session**

Perceived Confidence (7 Questions)

1. *My understanding of inguinal anatomy improved after today’s session.*
2. *I do not have further doubts/questions regarding inguinal anatomy.*
3. *I am confident with describing the layers of the inguinal canal.*
4. *I am confident with describing the differences between a direct VS indirect inguinal hernia.*
5. *I am confident in naming all the contents of the inguinal canal.*
6. *I feel more prepared in answering future exam questions related to inguinal anatomy.*
7. *When in the operating theatre, I feel that I can understand real-patient inguinal anatomy better after today’s session.*

Perceived Engagement (4 Questions)

1. *I found today’s session to be engaging.*
2. *I found today’s session to be fun.*
3. *I was able to maintain focus during today’s session.*
4. *I have a positive perception of the instructors who taught us.*

**Appendix 6 – Opinion on Model Creation Session**

Perception on Model Effectiveness (6 Questions)

1. *I felt that the model improved my ability to visualize the inguinal anatomy.*
2. *Creating the model helped me remember the inguinal anatomy better.*
3. *Creating the model helped to clarify doubts I had during the lecture.*
4. *Creating the model was an enjoyable experience.*
5. *I learned better through model creation than the lecture.*
6. *Creating the model felt more engaging than the lecture.*

Open Ended Responses (2 Questions)

1. *How could our lecture be improved?*
2. *How could our model creation session be improved?*
